# *Tropheryma whipplei*: A Cause of Rapidly Fatal Prosthetic Valve Endocarditis Despite a Classically Indolent Disease Course

**DOI:** 10.1155/cric/1675151

**Published:** 2026-09-12

**Authors:** Abdallatif Dawoud, Mohammad Safi, Maddy Taylor, Raghad Elhag, Oun Al-Dalahmeh, Omar Horani, Li Wang, Samer J. Khouri

**Affiliations:** ^1^ Department of Medicine, The University of Toledo, Toledo, Ohio, USA, utoledo.edu; ^2^ Division of Cardiovascular Medicine, The University of Toledo, Toledo, Ohio, USA, utoledo.edu; ^3^ Department of Medicine, University of Jordan, Amman, Jordan, ju.edu.jo; ^4^ ProMedica Toledo Hospital, Toledo, Ohio, USA

**Keywords:** culture-negative endocarditis, infective endocarditis, molecular diagnosis, periannular abscess, polymerase chain reaction, prosthetic valve endocarditis, *Tropheryma whipplei*

## Abstract

**Background:**

*Tropheryma whipplei* (*T. whipplei*) is a rare cause of culture‐negative infective endocarditis and may present without classic gastrointestinal manifestations, leading to delayed diagnosis and severe valvular destruction.

**Case Summary:**

A 76‐year‐old man with a prior bioprosthetic aortic valve replacement presented with at least 2 weeks of fatigue, generalized weakness, intermittent chills, and worsening dyspnea, along with approximately 8 kg of unintentional weight loss since the preceding summer; he remained afebrile. Blood cultures remained persistently negative. Transesophageal echocardiography revealed a > 3‐cm vegetation, prosthetic valve dehiscence, severe paravalvular regurgitation, and periannular abscess. Despite empiric broad‐spectrum antibiotics and urgent surgical evaluation, the patient deteriorated and suffered cardiac arrest. Whole‐blood polymerase chain reaction testing collected during hospitalization returned positive for *T. whipplei* after the patient′s death, establishing the diagnosis.

**Discussion:**

Whipple endocarditis typically presents as culture‐negative disease and often lacks systemic features, delaying recognition. This case highlights the diagnostic challenge posed by *T. whipplei* in prosthetic valve endocarditis and the value of molecular testing when conventional cultures remain negative.

**Take‐Home Message:**

Early PCR testing should be considered in culture‐negative prosthetic endocarditis, as it may establish a diagnosis when conventional cultures and valve tissue are unavailable.

**Take-Home Message**

*Tropheryma whipplei* (*T. whipplei*) should be considered in prosthetic valve culture‐negative endocarditis, particularly in afebrile patients with subacute presentations.

Early incorporation of PCR testing should be considered in culture‐negative endocarditis, as it may establish a diagnosis when conventional cultures and valve tissue are unavailable.

## 1. Case Presentation

### 1.1. Patient Presentation

A 76‐year‐old man—with a history of surgical bioprosthetic aortic valve replacement performed in August 2023 for severe aortic insufficiency with left ventricular systolic dysfunction and a possible sinus of Valsalva aneurysm, heart failure with preserved ejection fraction, chronic obstructive pulmonary disease, hypertension, hyperlipidemia, prior ischemic cerebrovascular accident, arthritis, obstructive sleep apnea on continuous positive airway pressure, and active tobacco use—presented approximately 2 years later with at least 2 weeks of fatigue, generalized weakness, and intermittent chills, as well as worsening dyspnea, approximately 8 kg (18 lbs) of unintentional weight loss since the preceding summer, and subsequent lower extremity edema. He was afebrile throughout his clinical course. Home medications included sacubitril/valsartan, carvedilol, spironolactone, dapagliflozin, furosemide, clopidogrel, and atorvastatin. He had no documented history of gastrointestinal disease, immunosuppression, malignancy, or chronic corticosteroid therapy. He was edentulous and denied recent dental procedures. His exposure history was notable for a reported possible exposure to a sewage treatment plant, although the nature, duration, and timing of the exposure were not documented.

On physical examination at presentation, the patient appeared ill and was hypoxemic, with an oxygen saturation of 92% on a 2‐L/min nasal cannula. Pulmonary examination revealed wheezing, and bilateral lower extremity edema was present. No peripheral signs of endocarditis were noted.

## 2. Diagnostic Assessment

### 2.1. Laboratory Findings

Initial laboratory evaluation demonstrated leukocytosis with neutrophilia (white blood cell count 14.5 × 10^9^/L on 1/13/26, transiently decreasing to 9.8 on 1/14 before rising to 13.9 on 1/15 and 16.1 on 1/16; absolute neutrophil count 11.6⟶7.7⟶12.0⟶15.1 × 10^9^/L), normocytic anemia (hemoglobin 12.0 → 11.3 → 10.9 → 11.2 g/dL; mean corpuscular volume 85–87 fL), and progressive thrombocytopenia (platelets 199⟶155⟶143⟶136 × 10^9^/L). Renal function remained preserved throughout hospitalization (creatinine 1.04 → 0.99 → 0.96 → 0.99 mg/dL). Electrolytes and liver function tests were otherwise unremarkable.

### 2.2. Inflammatory Markers

C‐reactive protein was markedly elevated at 7.2 mg/dL, whereas erythrocyte sedimentation rate was within normal limits at 16 mm/h.

### 2.3. Blood Cultures

Two sets of peripheral blood cultures were obtained on Hospital Day 2 during the same collection period, and both remained negative after 5 days of incubation.

## 3. Imaging

### 3.1. Transthoracic Echocardiography (TTE)

TTE demonstrated severe dysfunction of the bioprosthetic aortic valve, including severe aortic stenosis and severe aortic regurgitation, with preserved left ventricular systolic function (estimated ejection fraction 50%–55%). These findings raised concern for prosthetic valve pathology and prompted further diagnostic evaluation.

### 3.2. Transesophageal Echocardiography (TEE)

**Figure 1:**
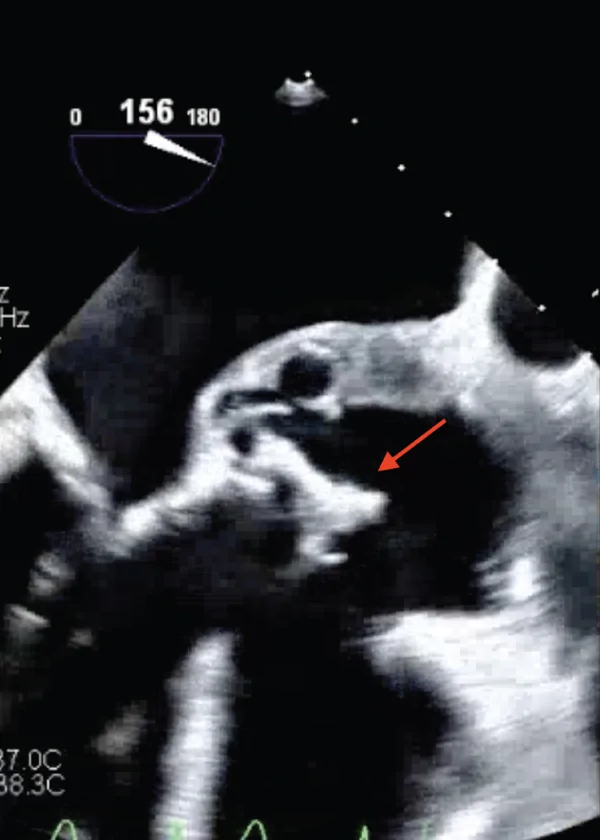
Transesophageal echocardiography demonstrating a large, multilobulated, mobile echogenic mass attached to the bioprosthetic aortic valve, consistent with vegetation.

**Figure 2:**
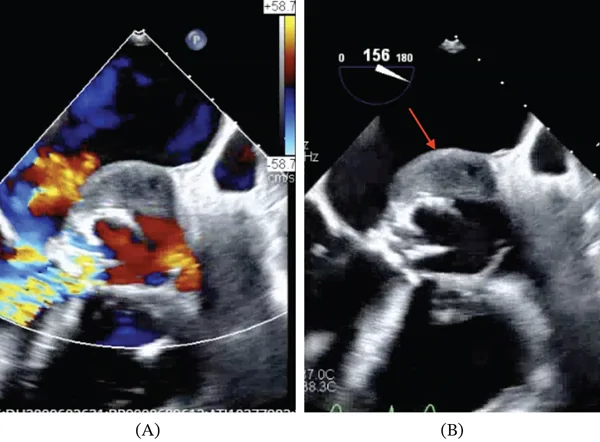
(A) Color Doppler transesophageal echocardiography demonstrating a large eccentric paravalvular regurgitant jet originating adjacent to the bioprosthetic valve annulus, consistent with prosthetic valve dehiscence and periannular destruction. (B) Transesophageal echocardiography demonstrating distortion of the bioprosthetic valve architecture with annular involvement, concerning for periannular abscess and prosthetic instability.

TEE performed at our institution (Video S1) revealed extensive prosthetic valve involvement, including a large (> 3 cm) echogenic mass consistent with vegetation on the bioprosthetic aortic valve (Figure 1). The prosthesis demonstrated dehiscence, consistent with mechanical instability, and was associated with severe perivalvular aortic regurgitation (Figure 2A). A complex periaortic abscess was identified, with evidence of fistulous or pseudoaneurysm formation extending into the periannular region (Figure 2B). The vegetation was characterized as high risk for embolization. Additional findings included moderate‐to‐severe mitral regurgitation, raising concern for possible multivalvular involvement.

## 4. Diagnostic Challenges and Differential Diagnosis

This case was diagnostically challenging because advanced, destructive prosthetic valve endocarditis was present despite persistently negative routine blood cultures. Two sets of peripheral blood cultures were obtained on Hospital Day 2 during the same collection period, and both remained negative after 5 days of incubation. Early recognition was further complicated by the patient′s subacute, afebrile presentation, which included at least 2 weeks of fatigue, generalized weakness, and intermittent chills, along with unintentional weight loss since the preceding summer.

Transesophageal echocardiographic findings were strongly suggestive of infective endocarditis despite negative cultures.

Potential explanations for blood culture–negative endocarditis (BCNE) included prior antimicrobial exposure; infection with fastidious or intracellular organisms, including *Bartonella* species, *Coxiella burnetii*, *Brucella* species, and *T. whipplei*; fungal infection; and, less likely, noninfectious etiologies such as nonbacterial thrombotic endocarditis. The infectious diseases team considered the reported possible exposure to a sewage treatment plant when selecting additional diagnostic tests, although the nature, duration, and timing of the exposure were not documented. Whole‐blood polymerase chain reaction testing for *T. whipplei* was therefore included in the expanded BCNE evaluation and was performed using a laboratory‐developed assay developed and performed by Mayo Clinic Laboratories (Rochester, Minnesota, United States). The broader evaluation also included *Bartonella* serologies and fungal biomarkers. Testing for *C. burnetii* and *Brucella* species was considered in the infectious diseases plan but was not obtained before the patient′s rapid clinical deterioration.

The *Bartonella* antibody panel demonstrated a *Bartonella henselae* IgG titer of 1:256 with IgM < 1:20; *Bartonella quintana* IgG was < 1:128 and IgM was < 1:20. This serologic pattern was not definitive for active *Bartonella* endocarditis. The fungal evaluation was unrevealing: Histoplasma mycelial and yeast complement‐fixation titers were < 1:8; histoplasma precipitin antibodies were not detected; histoplasma/blastomyces antigen was not detected; serum cryptococcal antigen was negative; *Aspergillus* galactomannan was < 0.500; and serum *β*‐D‐glucan was < 31 with a negative qualitative result. Whole‐blood PCR for *T. whipplei*, collected during hospitalization, subsequently returned positive after the patient′s death. At the time the PCR result became available, several *Bartonella* and fungal send‐out studies remained pending, and planned testing for *C. burnetii* and *Brucella* species had not been obtained; therefore, the BCNE evaluation had not been fully completed, although no other definitive etiology was ultimately established.

## 5. Therapeutic Intervention

After two sets of peripheral blood cultures were obtained, the infectious diseases service initiated empiric therapy with ceftriaxone 2 g intravenously every 24 h. Vancomycin was initially ordered but was not administered; daptomycin 500 mg intravenously every 24 h was recommended instead to provide broader enterococcal coverage, given the patient′s history of urinary dribbling and concern for a possible occult urinary source of enterococcal bacteremia and prosthetic valve endocarditis. The treatment strategy was to maintain broad empiric antimicrobial coverage while pursuing an expanded evaluation for culture‐negative endocarditis, including repeat blood cultures and serologic and molecular testing for fastidious pathogens.

Given the presence of large vegetations, periannular abscess formation, prosthetic valve dehiscence, and progressive hemodynamic instability, urgent surgical intervention was recommended. Cardiothoracic surgery evaluated the patient for redo aortic valve replacement, with possible reconstruction of the aortomitral curtain because of the root abscess and annular involvement. However, definitive surgical management could not be pursued prior to clinical deterioration.

## 6. Outcomes

**Figure 3:**
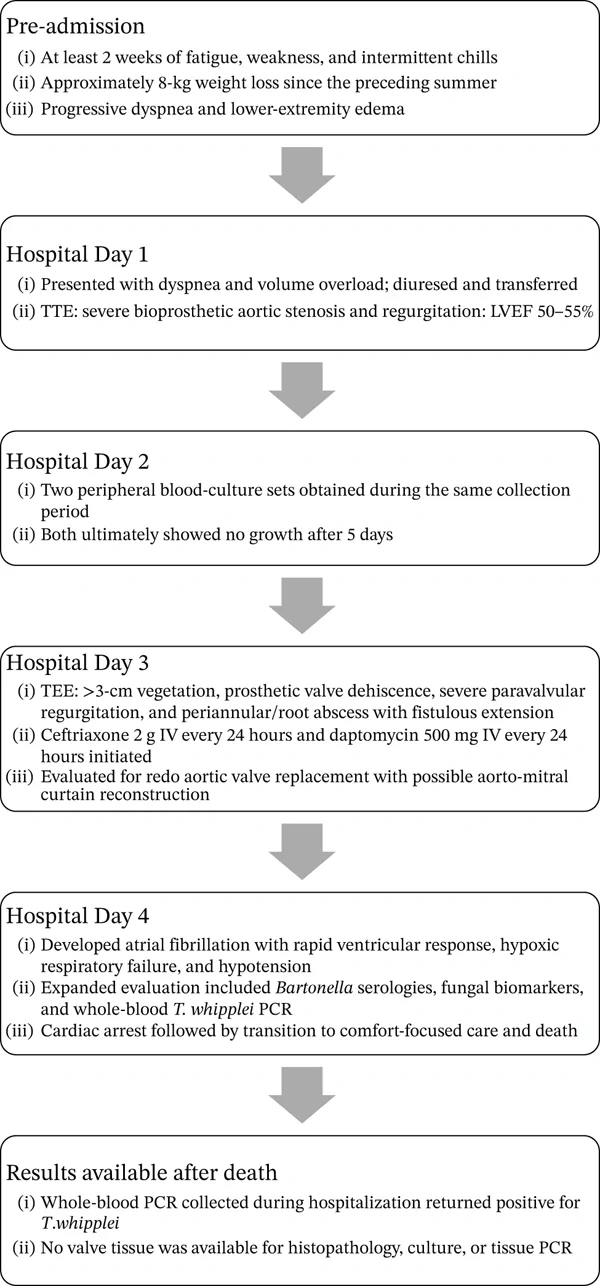
Clinical timeline of presentation, diagnostic evaluation, antimicrobial therapy, and outcome in a patient with *Tropheryma whipplei* prosthetic valve endocarditis.

During hospitalization, the patient experienced rapid clinical decline, including new‐onset atrial fibrillation with rapid ventricular response, worsening hypoxic respiratory failure, and progressive hypotension. Despite the escalation of medical therapy and multidisciplinary care, he suffered cardiac arrest. After discussion with the family and in accordance with the patient′s previously expressed wishes, resuscitative efforts were discontinued and care was transitioned to a comfort‐focused approach. The patient died shortly thereafter. Blood PCR testing for *T. whipplei*, sent during hospitalization as part of the culture‐negative endocarditis evaluation, later returned positive after the patient′s death, establishing the diagnosis. A detailed timeline of the patient′s presentation, diagnostic workup, and clinical course is summarized in Figure 3.

## 7. Discussion

Whipple disease is caused by *T. whipplei*, an intracellular organism widely distributed in soil and sewage systems. Despite its ubiquity, disease remains uncommon, likely because most individuals clear the organism efficiently [1]. Although classically associated with arthralgias (70%–90%) and gastrointestinal symptoms—most commonly diarrhea (70%–85%) and weight loss (80%–90%)—resembling malabsorption syndromes [2], these features may be absent in the endocarditis subtype, which often follows a prolonged subclinical course due to slow macrophage infiltration of affected tissues [3, 4].

In Whipple endocarditis, cardiac valves are primarily involved, frequently without gastrointestinal manifestations [3, 5]. The Spanish GAMES study evaluated a cohort of 3165 endocarditis cases and identified that only 14.2% were diagnosed with BCNE, with 3.5% of these found to have *T. whipplei* endocarditis, emphasizing how rare the diagnosis is [6]. One study has also described notable geographic variation in incidence among BCNE cases, ranging from under 3% to over 7% depending on the country studied [7]. A systematic review of 127 reported cases found prosthetic valve involvement in 8%, most commonly affecting the aortic valve. Fever occurred in less than 30% of patients, and diagnosis was established by valve PCR or histology in 88.2% of cases. Reported mortality was 9.4% [8]. Although data specific to Whipple endocarditis is limited, paravalvular extension is a documented complication in endocarditis and can occur in roughly one‐fifth of cases (21.7%), with complete heart block present in 8% and an operative mortality rate of 16% [9].

Whipple endocarditis presents as BCNE [1]. Causes of BCNE include prior antibiotic exposure, infection with fastidious or intracellular organisms, and noninfectious etiologies. *T. whipplei* belongs to the fastidious intracellular group, alongside *C. burnetii*, *Bartonella* species, and *Brucella* species [5]. Because it does not grow in routine blood cultures, molecular diagnostic techniques, particularly PCR testing, are required for diagnosis [5]. The disease typically follows an indolent course, and patients are frequently afebrile, contributing to delayed recognition, often after significant valvular damage has occurred and diagnosis is established through PCR of surgically obtained valve tissue [10]. Arthralgias or arthritis commonly precede cardiac involvement and may represent the predominant extracardiac manifestation in the absence of overt infectious signs [10].

PCR can detect *T. whipplei* from tissue or blood, and its identification is now incorporated as a major microbiologic criterion in the 2023 Duke‐ISCVID criteria [5, 11]. Blood PCR sensitivity remains low (31.2%), meaning negative results do not rule out the diagnosis [7]. However, in the present case, the presence of other major imaging criteria (vegetation, dehiscence, periannular abscess) along with positive blood PCR fulfills the definition of definitive endocarditis according to the 2023 Duke ISCVID criteria [5, 11].

The suggested diagnostic algorithm, per the American Heart Association (AHA), for BCNE starts with obtaining a preantibiotic sample for future metagenomic sequencing, particularly when BCNE is suspected, followed by obtaining ≥ 2 sets of cultures under strict aseptic technique. If sets remain negative after 72 h, testing is expanded to include serology for *C. burnetii*, *Bartonella species*, and *Brucella* species, along with whole blood PCR for *C. burnetii*, *Bartonella species*, and *T. whipplei* [5]. Separately, when surgery is indicated, the excised tissue should be sent for Gram stain and culture, PCR (including for *T. whipplei*, *C. burnetii*, and *Bartonella* species, among other fastidious organisms), amplicon or metagenomic sequencing, and immunohistochemistry [5]. In the present case, surgery was indicated but could not be done owing to the rapid clinical deterioration of this patient. The PCR workup sent during hospitalization, as part of the culture‐negative endocarditis workup, returned positive for *T. whipplei* only after the patient′s death, precluding tissue‐based diagnosis.

Interestingly, the present case has an elevated CRP but a normal ESR—further evidence of how inconsistent and atypical the lab findings can be in this patient′s presentation, compounding the diagnostic challenge already posed by persistently negative blood cultures and an afebrile, subacute presentation.

Treatment of Whipple endocarditis requires prolonged antimicrobial therapy. Standard management includes an initial induction phase of 2–4 weeks with intravenous penicillin or ceftriaxone, followed by long‐term oral therapy to prevent relapse. Maintenance regimens may include trimethoprim–sulfamethoxazole or doxycycline combined with hydroxychloroquine, often extended for 1 year or longer [5].

## 8. Conclusion

This case adds to the limited literature on *T. whipplei* endocarditis by demonstrating that severe, destructive prosthetic valve infection can occur in an immunocompetent, afebrile patient without gastrointestinal manifestations. The subacute presentation and persistently negative blood cultures illustrate the diagnostic challenge and the potential for delayed recognition to contribute to valvular destruction and poor outcomes in general. Importantly, this case underscores the need to maintain clinical suspicion for *T. whipplei* even in the absence of classic systemic features and highlights the critical role of early molecular testing. Blood PCR helped establish the diagnosis when surgical tissue was unavailable. Earlier identification of *T. whipplei* would not have altered the timing of surgical intervention, as major structural indications were already present on echocardiography. Moreover, the patient was already receiving ceftriaxone, an agent with activity against *T. whipplei*, as part of empiric therapy. Whether earlier diagnosis would have changed the prognosis remains uncertain given the severity of valvular destruction and rapid clinical deterioration.

## Funding

No funding was received for this manuscript.

## Ethics Statement

This case report was conducted in accordance with the ethical standards of the institutional and national research committee and with the Declaration of Helsinki. Institutional Review Board approval was not required for a single case report in accordance with institutional policy

## Consent

The patient is deceased, and verbal informed consent for publication of this case report was obtained from the patient′s legally authorized representative. All reasonable efforts have been made to protect patient anonymity.

## Conflicts of Interest

The authors declare no conflicts of interest.

## Supporting information


**Supporting Information** Additional supporting information can be found online in the Supporting Information section. Video S1: Transesophageal echocardiography demonstrates extensive prosthetic aortic valve endocarditis, with a large (>3 cm) echogenic vegetation involving the bioprosthetic valve. The prosthesis shows partial dehiscence with severe perivalvular aortic regurgitation, along with a complex periaortic abscess and extension into the periannular region with fistulous or pseudoaneurysmal formation.

## Data Availability

The data that support the findings of this study are available on request from the corresponding author. The data are not publicly available due to privacy or ethical restrictions.
