# Supplementary material for: Tropheryma whipplei: A Cause of Rapidly Fatal Prosthetic Valve Endocarditis Despite a Classically Indolent Disease Course
Source: Case Rep Cardiol. 2026 Sep 12;2026:1675151. doi: 10.1155/cric/1675151 (PMC13570416; doi:10.1155/cric/1675151)
Supplement: Supplementary file 1 [file CRIC-2026-1675151-s001.docx]

Video 1 showed extensive prosthetic valve involvement, including a large (>3 cm) echogenic mass consistent with vegetation on the bioprosthetic aortic valve. The prosthesis demonstrated dehiscence, consistent with mechanical instability, and was associated with severe perivalvular aortic regurgitation. A complex periaortic abscess was identified, with evidence of fistulous or pseudoaneurysm formation extending into the periannular region. The vegetation was characterized as high risk for embolization. Additional findings included moderate-to-severe mitral regurgitation, raising concern for possible multivalvular involvement
